# Post-transplant cyclophosphamide separates graft-versus host disease and graft versus leukemia effects after HLA-matched stem-cell transplantation for acute myeloid leukemia

**DOI:** 10.1038/s41375-024-02445-x

**Published:** 2024-10-31

**Authors:** Avichai Shimoni, Christophe Peczynski, Myriam Labopin, Alexander Kulagin, Ellen Meijer, Jan Cornelissen, Goda Choi, Jaime Sanz, Montserrat Rovira, Gwendolyn Van Gorkom, Nicolaus Kröger, Yener Koc, Jan Vydra, J. L. Diez-Martin, Carlos Solano, Amit Patel, Patrizia Chiusolo, Fabio Ciceri, Arnon Nagler, Mohamad Mohty

**Affiliations:** 1 Division of Hematology, Chaim Sheba Medical Center, Tel-Hashomer and Tel-Aviv University, Tel Aviv, Israel; 2 Sorbonne University, INSERM UMRs 938, Paris, France; 3 Gorbacheva Research Institute, Pavlov University, St. Petersburg, Russia; 4 VU University Medical Center, Department of Hematology, Amsterdam, The Netherlands; 5 Erasmus MC Cancer Institute, University Medical Center Rotterdam, Department of Hematology, Rotterdam, The Netherlands; 6 University Medical Center Groningen (UMCG), Dept. of Hematology, Groningen, The Netherlands; 7 University Hospital La Fe, Hematology Department, Valencia, Spain; 8 Hospital Clinic, Institute of Hematology & Oncology, Dept. of Hematology, Barcelona, Spain; 9 University Hospital Maastricht, Dept. Internal Med.Hematology /Oncology, Maastricht, The Netherlands; 10 University Hospital Eppendorf, Bone Marrow Transplantation Centre, Hamburg, Germany; 11 Medicana International Hospital Istanbul, Bone Marrow Transplant Unit, Istanbul, Turkey; 12 Institute of Hematology and Blood Transfusion, Servicio de Hematología, Prague, Czech Republic; 13 Hospital Gregorio Marañón, Sección de Trasplante de Medula Osea, Madrid, Spain; 14 Hospital Clínico de Valencia, Servicio de Hematología, Valencia, Spain; 15 Clatterbridge Cancer Centre - Liverpool, Royal Liverpool University Hospital, Clatterbridge Cancer Centre NHS Foundation Trust, Division of Stem Cell Transplantation and Haemato., Liverpool, United Kingdom; 16 Universita Cattolica S. Cuore, Istituto di Ematologia, Ematologia, Rome, Italy; 17 Hematology and Bone Marrow Transplant Unit, San Raffaele Scientific Institute, Milan, Italy; 18 Hématologie Clinique et Thérapie Cellulaire, Hôpital Saint-Antoine, Paris, France

**Keywords:** Acute myeloid leukaemia, Bone marrow transplantation

## Abstract

The association of graft-versus-host disease (GVHD) and graft-versus-leukemia (GVL) effects after allogeneic stem-cell transplantation (SCT) is well-established but was not confirmed in the modern era and following post-transplant cyclophosphamide (PTCy). We assessed GVHD/ GVL association in AML patients following HLA-matched SCT with standard calcineurin-based (*n* = 12,653, 57% with additional in-vivo T-cell depletion) or PTCy-based (*n* = 508) GVHD prophylaxis. Following standard prophylaxis, acute GVHD grade II-IV and III-IV, chronic GVHD, and extensive chronic GVHD rates were 23.8%, 7.5%, 37.0%, and 16.3%, respectively. Acute GVHD grade II and III-IV were associated with lower relapse [hazard-ratio (HR) 0.85, *P* = 0.002; HR 0.76, *P* = 0.003, respectively)], higher non-relapse mortality (NRM) (HR 1.5, *P* < 0.001; HR 6.21, *P* < 0.001) and lower overall survival (OS) (HR 1.49, *P* < 0.001; HR 6.1, *P* < 0.001). Extensive chronic GVHD predicted lower relapse (HR 0.69, *P* < 0.001), higher NRM (HR 2.83, *P* < 0.001), and lower OS (HR 2.74, *P* < 0.001). Following PTCy, GVHD rates were 22.8%, 6.2%, 35.5%, and 17.7%, respectively. Acute GVHD was not associated with relapse (HR 1.37, *P* = 0.15) but predicted higher NRM (HR 3.34, *P* < 0.001) and lower OS (HR 1.92, *P* = 0.001). Chronic GVHD was not prognostic for these outcomes. In conclusion, GVHD and GVL are strongly associated with contemporary SCT. However, following PTCy, GVHD is not associated with reduced relapse.

## Introduction

Allogeneic stem cell transplantation (SCT) is a curative therapy for acute myeloid leukemia (AML). It provides both dose-intensive chemo-radiotherapy and induction of a graft-versus leukemia effect (GVL). The most significant cause of treatment-related toxicity is graft-versus-host disease (GVHD). These two immune effects, GVHD and GVL, are closely associated and survival after SCT is dependent on the balance between their opposing effects on outcome. This association was well established in the early era of SCT [1–3]. Horowitz et al. have shown that both acute and chronic GVHD are associated with a lower risk for relapse after SCT [3]. However, only the mild forms were associated with better survival as the more severe GVHD forms also resulted in increased non-relapse mortality (NRM). Chronic GVHD was more important in controlling relapse in patients with AML.

Marked changes have been introduced in modern SCT over the last two decades. These include SCT in older and less fit patients, the use of reduced-intensity conditioning (RIC), the use of older sibling donors, more unrelated donors, as well as alternative donors such as haploidentical and umbilical cord blood donors. In addition, there was a change to a more common use of peripheral blood stem cells (PBSC) as the stem-cell source as well as post-transplant interventions to reduce the risk of relapse. Better supportive care and control of infections have reduced NRM. Novel approaches for GVHD prophylaxis and treatment have also been introduced. The combination of a calcineurin inhibitor and methotrexate has been the backbone of GVHD prophylaxis for several decades [4]. Post-transplant cyclophosphamide (PTCy) was more recently introduced for GVHD prophylaxis in non-T-cell depleted haploidentical SCT and later also in the HLA-matched setting [5–9]. These changes resulted in markedly improved NRM and survival after SCT but to a much lesser extent, lower risk of disease relapse [10].

All of these changes may have had a theoretical impact on the association of GVHD and GVL in the more contemporary SCT era. A more recent analysis of the correlation between relapse and GVHD in a mega-file of more than 48,000 transplants reported to the European Society for Blood and Marrow Transplantation (EBMT) confirmed the well-known association of GVHD and GVL [11]. However, the strength of the association was relatively weak in patients with AML [11]. Several studies have shown that the role of GVL in AML was more important after RIC than after myeloablative conditioning (MAC) [12, 13].

The use of haploidentical SCT has markedly increased over the last decade with outcomes that have constantly improved with time, approaching those of unrelated donor transplants [14]. This is mostly related to the shift towards non-T-cell depleted transplants with the use of PTCy. Studies of the Acute Leukemia Working Party (ALWP) of the EBMT showed that in the haploidentical SCT setting with PTCy, there was no association between GVHD and GVL [15, 16]. GVHD of any form or grade did not improve the relapse rate after haploidentical SCT while the severe forms increased NRM and reduced survival. However, these studies could not determine if this observation was related to the haploidentical SCT setting or to the use of PTCy.

In the current study, we assessed the association of GVHD and GVL in two separate groups of AML patients according to the GVHD prophylaxis group. The first group was a large cohort of AML patients given SCT in the contemporary era with standard GVHD prophylaxis. Here the goal was to assess the occurrence and strength of the GVHD/GVL association in the modern era of SCT. The second smaller group consisted of AML patients given PTCy after HLA-matched transplants. This was aimed to further explain the lack of association between GVHD and GVL we observed in patients with AML following haploidentical SCT with PTCy. The study was not designed to compare the two groups of GVHD prevention.

## Patients and Methods

### Study design and data collection

This is a retrospective multicenter analysis. Patient data were obtained from the EBMT registry. The EBMT is an international research collaborative group comprising over 650 transplant centers required to report on an annual basis on all transplants performed. Quality control measures of this multicenter registry include confirmation of the validity of the entered data by the reporting team, cross-checking with the national registries, and regular in-house and external data audits. The study was approved by the ALWP and was performed in compliance with the Declaration of Helsinki and under the guidance of the EBMT. All patients provided written informed consent authorizing the use of information for research purposes.

Patients were eligible for the study if they were more than 18 years old, had de-novo AML in first complete remission (CR1) or second complete remission (CR2), and received a first allogeneic SCT from an HLA-matched donor between the years 2010-2019. Only HLA- matched sibling and 10/10 matched-unrelated donor transplants were included. Both bone marrow (BM) and PBSC were eligible stem cell sources. Mismatched unrelated, haploidentical, and umbilical cord blood transplants were excluded from this study. Data collected included recipient and donor characteristics, disease features, transplant-related factors including the conditioning regimen, type of GVHD prophylaxis, and outcome variables including the occurrence and timing of acute and chronic GVHD, relapse, and survival data. The conditioning regimen was reported at the participating center’s discretion. Dose intensity was defined according to standard EBMT criteria [17].

### GVHD prophylaxis

The GVHD prophylaxis regimen was reported according to the participating center policy. Two separate analyses were carried on based on the GVHD prophylaxis regimen. The first analysis was focused on a standard GVHD prophylaxis regimen which was based on a calcineurin inhibitor (cyclosporine A or tacrolimus) with short course methotrexate or mycophenolate mofetil in most cases (Table 1). The second analysis focused on PTCy with or without additional GVHD prophylaxis agents. Patients in the standard prophylaxis analysis could have been given anti-thymocyte globulin (ATG) or alemtuzumab. Patients in the PTCy analysis who were given additional in-vivo T-cell depletion such as ATG or alemtuzumab were excluded. Patients having ex-vivo T-cell depletion were excluded from both cohorts.

**Table 1:** Patient and Transplant Characteristics.

Patient Characteristics		
GVHD prophylaxis	Standard	PTCy
Total number	12653	508
Median age, years (range)	52 (18–80)	49 (18–72)
Gender; Male	6688 (53%)	281 (55%)
Female	5943 (47%)	226 (45%)
Median year of SCT	2014 (2010–2019)	2017 (2010–2019)
Donor; Sibling	6726 (53%)	234 (46%)
MUD	5927 (47%)	274 (54%)
Stem cell source; BM	1399 (11%)	92 (18%)
PBSC	11254 (89%)	416 (82%)
Status at SCT; CR1	10478 (83%)	437 (86%)
CR2	2175 (17%)	71 (14%)
Cytogenetics; Good	792 (6%)	32 (6%)
Intermediate	5626 (45%)	272 (54%)
Adverse	1761 (14%)	76 (15%)
Unknown	4474 (35%)	128 (25%)
F → M	2225 (18%)	99 (20%)
KS; ≥ 90	9553 (80%)	374 (76%)
< 90	2383 (20%)	117 (24%)
Conditioning; RIC	5711 (45%)	215 (42%)
MAC	6942 (55%)	293 (58%)
GVHD prophylaxis;		
CNI alone	1844 (15%)	151 (30%)^a^
CNI/ MTX	6790 (53%)	51 (10%)^a^
CNI/ MMF	3339 (26%)	173 (34%)^a^
Other	680 (5%)	64 (13%)^a^
None	0 (0%)	69 (14%)^a^
In vivo T-cell depletion; (ATG/ alemtuzumab)	7165 (57%)	0 (0%)
**Transplantation Outcomes**		
2-year Overall Survival	68.8% [67.9–69.6]	69.7% [64.9–74.9]
2-year Leukemia-free survival	61.8% [60.9–62.7]	63.6% [58.7–68.9]
2-year Relapse incidence	26.4% [25.6–27.3]	27.1% [22.6–31.9]
2-year Non-relapse mortality	11.8% [11.2–12.4]	9.3% [6.6–12.5]
Acute GVHD (day 180)		
Grade II-IV	23.8% [23–24.5]	22.8% [19.2–26.6]
Grade III-IV	7.5% [7.1–8.0]	6.2% [4.3–8.6]
Chronic GVHD (2 years)		
All grades	37% [36.1–37.9]	35.5% [30.7–40.2]
Extensive	16.3% [15.7–17]	17.7% [14.0–21.7]
GVHD-free, relapse-free survival	47.8% [46.9–48.8]	49.1% [44.2–54.5]

*GVHD* graft-versus-host disease, *PTCy* post-transplant cyclophosphamide, *SCT* stem cell transplantation, *MUD* matched unrelated donor, *BM* bone marrow, *PBSC* peripheral blood stem cells, *CR* complete remission, *F→M* female donor to male recipient, *KS* Karnofsky score, *RIC* reduced intensity conditioning, *MAC* myeloablative conditioning, *CNI* calcineurin inhibitor, *MTX* methotrexate, *MMF* mycophenolate mofetil. ^a^In addition to PTCy.

### Evaluation of outcomes

Overall survival (OS) was calculated from the day of SCT until death from any cause or the date of last follow-up. Leukemia-free survival (LFS) was defined as survival with no relapse. Disease relapse was defined according to standard hematological criteria. NRM was defined as death without prior disease recurrence. Acute GVHD was graded and staged according to the Consensus criteria [18]. Chronic GVHD was graded according to the Seattle criteria [19].

### Statistical analysis

The primary endpoint of the study was relapse incidence after SCT. Secondary endpoints were NRM, LFS, and OS. All analyses were performed separately for the group of patients given standard GVHD prophylaxis regimen without PTCy and the group given PTCy. The study was not designed to compare these 2 groups, but rather to evaluate the association of GVHD and GvL in each cohort separately. The probabilities of OS and LFS were calculated using the Kaplan–Meier method [20]. Relapse incidence, NRM, and acute and chronic GVHD incidences were estimated using cumulative incidence analysis, considering competing risks. In the estimation of acute and chronic GVHD, we considered relapse and death to be competing events [21]. Baseline characteristics of patient, disease, and transplantation procedures were described as frequency and percentage for qualitative variables and as median and interquartile range (IQR) for quantitative variables. To study the effect of GVHD on SCT outcomes, we used Cox proportional hazards models including acute and chronic GVHD as time-dependent variables. The Cox proportional hazards assumption was verified using both cox.zph function (survival package in R) and graphs of Schoenfeld residuals for all outcomes. The following covariates were also included in the multivariate models for adjustment: year of transplantation (continuous), cell source (bone marrow vs. peripheral blood), type of donor (identical sibling vs. matched unrelated donor 10/10), status at transplant (CR1 vs. CR2), AML cytogenetics (good/intermediate/unknown vs. poor), patient age (continuous), female to male combination (female to male vs. other), Karnofsky score (>=90 vs. <90) and intensity of conditioning (RIC vs. MAC). These variables were selected because of known clinical relevance and/or impact in univariate analysis. Hazard ratios (HR) and 95% confidence intervals (95% CI) are reported. The center effect was taken into account by introducing a random effect or ‘frailty’ into all models. The interpretation of the results is based on the results of multivariate analyses where p values are given versus a reference group which is, for acute GVHD, the absence of acute GVHD or acute GVHD grade I, and for chronic GVHD, the absence of any chronic GVHD. In the standard prophylaxis without PTCy cohort, the time-dependent effect of acute GVHD was divided in two modalities: grade II and grade III-IV. Chronic GVHD was also divided into two modalities: limited CGVHD and extensive CGVHD. As the number of events of acute GVHD grade III-IV and extensive chronic GVHD was too low in the PTCy cohort, the two aforementioned modalities were combined in one modality: acute GVHD grade II-IV and chronic GVHD (whatever the extent). Differences were considered statistically significant in the case of *P* < 0.05. Statistical analyses were performed with R 4.1.2 (R Core Team (2017). R: A language and environment for statistical computing. R Foundation for Statistical Computing, Vienna, Austria. URL https://www.R-project.org/.).

## Results

### Standard GVHD prophylaxis cohort

#### Patient and transplant characteristics

This part of the study included 12,653 adult patients with de-novo AML in CR1 or CR2 given a first allogeneic SCT from an identical sibling donor or a 10/10 matched unrelated donor between the years 2010 and 2019. Patient and transplant characteristics are shown in Table 1. The median patient age was 52 years. The conditioning regimen was myeloablative in 55% and RIC in 45%. GVHD prophylaxis in this cohort included a calcineurin inhibitor either alone or with a short course of methotrexate or mycophenolate mofetil in 95% of patients.

#### Transplantation outcomes

Transplantation outcomes are presented in Table 1. The incidence of acute GVHD grade II-IV and III-IV at day +180 after SCT was 23.8% (95% CI, 23–24.5) and 7.5% (95% CI, 7.1–8.0), respectively. The incidence of all grades and extensive grade chronic GVHD at 2 years after SCT was 37.0% (95% CI, 36.1–37.9) and 16.3% (95% CI, 15.7–17), respectively. The 2-year OS was 68.8% (95% CI, 67.9–69.6). The incidence of relapse and NRM at 2 years after allogeneic SCT was 26.4% (95% CI, 25.6–27.3) and 11.8% (95% CI, 11.2–12.4), respectively.

#### Impact of GvHD on survival outcomes

The Cox multivariate model showed that acute GVHD grade II was associated with a lower incidence of relapse (HR 0.85, *P* = 0.002, Table 2), higher NRM, (HR 1.5, *P* < 0.001), and lower OS (HR 1.49, *P* < 0.001). Acute GVHD grade III-IV was also associated with a lower incidence of relapse (HR 0.76, *P* = 0.003, Table 2), higher NRM, (HR 6.21, *P* < 0.001), and lower OS (HR 6.1, *P* < 0.001).

**Table 2:** Prognostic factors for SCT outcomes after standard GVHD prophylaxis.

	Relapse		NRM		OS	
Factor	HR	*P* value	HR	*P* value	HR	*P* value
Acute GVHD						
grade II	0.85 [0.77–0.94]	0.002	1.5 [1.31–1.72]	<0.001	1.49 [1.3–1.7]	<0.001
grade III-IV	0.76 [0.64–0.91]	0.003	6.21 [5.48–7.03]	<0.001	6.1 [5.39–6.9]	<0.001
Chronic GVHD						
limited	0.97 [0.86–1.09]	0.56	1.15 [0.96–1.37]	0.14	1.14 [0.95–1.36]	0.15
extensive	0.69 [0.59–0.81]	<0.001	2.83 [2.46–3.25]	<0.001	2.74 [2.39–3.15]	<0.001
Age	1 [1–1]	0.77	1.04 [1.03–1.04]	<0.001	1.04 [1.03–1.04]	<0.001
Year of SCT	1 [0.99–1.02]	0.86	0.95 [0.93–0.97]	<0.001	0.96 [0.94–0.98]	<0.001
Donor (MUD)	0.88 [0.82–0.96]	0.002	1.25 [1.11–1.39]	0.001	1.22 [1.09–1.36]	<0.001
Stem cell source (PBSC)	0.98 [0.87–1.09]	0.69	1.1 [0.92–1.32]	0.28	1.13 [0.94–1.34]	0.19
Status at SCT (CR2)	1.23 [1.12–1.35]	<0.001	1.19 [1.05–1.35]	0.008	1.2 [1.06–1.36]	0.004
Cytogenetics (adverse)	1.83 [1.68–2]	<0.001	1.19 [1.02–1.38]	0.025	1.22 [1.06–1.41]	0.007
Gender combination (other than F→M)	1.11 [1.01–1.23]	0.026	0.75 [0.66–0.84]	<0.001	0.75 [0.67–0.85]	<0.001
KS (<90)	1.03 [0.94–1.12]	0.53	1.22 [1.08–1.37]	0.001	1.19 [1.06–1.34]	0.004
Conditioning (MAC)	0.92 [0.85–1]	0.052	1.19 [1.06–1.34]	0.004	1.19 [1.06–1.34]	0.003
In vivo T-cell depletion (no ATG/ alemtuzumab)	0.98 [0.91–1.07]	0.7	1.02 [0.91–1.15]	0.68	1.02 [0.91–1.14]	0.7

Multivariate analysis using acute GVHD and chronic GVHD as time-dependent factors. Abbreviations as in Table 1. *HR* hazard ratio.

The Cox multivariate model showed that extensive chronic GVHD was associated with a lower incidence of relapse (HR 0.69, *P* < 0.001, Table 2), higher NRM, (HR 2.83, *P* < 0.001) and lower OS (HR 2.74, *P* < 0.001). Limited Chronic GVHD was not associated with any of these transplantation outcomes (Table 2).

The same association between acute and chronic GVHD and transplantation outcome was seen in the subgroup of patients given in-vivo T-cell depletion and those who did not (Table 3). For example among patients given T-cell depletion acute GVHD grade II-IV and chronic GVHD were associated with lower relapse rates (HR 0.86 *P* = 0.018 and HR 0.75, *P* < 0.001, respectively, Table 3). Similarly among patients not given T-cell depletion acute GVHD grade II-IV and chronic GVHD were also associated with lower relapse rates (HR 0.81 *P* = 0.002 and HR 0.63, *P* < 0.001, respectively, Table 3). There were no statistically significant interactions between acute and chronic GvHD and cell source or cytogenetics on all outcomes to merit further subgroup analysis.

**Table 3:** Prognostic factors for SCT outcomes after standard GVHD prophylaxis by use of in-vivo depletion.

	Relapse		NRM		OS	
Patients treated with ATG or Campath						
Factor	HR	*P* value	HR	*P* value	HR	*P* value
Acute GVHD						
grade II–IV	0.86 [0.76-0.97]	0.018	2.56 [2.22–2.96]	<0.001	1.54 [1.39–1.69]	<0.001
Chronic GVHD	0.75 [0.65–0.86]	<0.001	2.11 [1.78–2.49]	<0.001	0.88 [0.79–0.98]	0.024
**Patients not treated with ATG or Campath**						
Acute GVHD						
grade II-IV	0.81 [0.71–0.93]	0.002	2.8 [2.4–3.27]	<0.001	1.47 [1.32–1.63]	<0.001
Chronic GVHD	0.63 [0.54–0.74]	<0.001	1.81 [1.49–2.19]	<0.001	0.71 [0.63–0.79]	<0.001

Multivariate analysis using acute GVHD and chronic GVHD as time dependent factors. As in Tables 1–2. HR, hazard ratio.

The multivariate analysis identified advanced age (HR 1.04, *P* < 0.001), matched unrelated donor (HR 1.22, *P* < 0.001), CR2 compared to CR1 (HR 1.20, *P* = 0.004), adverse cytogenetics (HR 1.22, *P* = 0.007), female donor to male recipient (*P* < 0.001), lower Karnofsky score (HR 1.19, *P* = 0.004) and MAC regimen (HR 1.19, *P* = 0.003) as poor prognostic factors for OS, in addition to the poor prognosis associated with acute GVHD and extensive chronic GVHD (Table 2).

### PTCy prophylaxis cohort

#### Patient and Transplant Characteristics

This analysis included 508 adult patients with de-novo AML in CR1 or CR2 given a first allogeneic SCT from an identical sibling donor or a 10/10 matched unrelated donor between the years 2010 and 2019. Patient and transplant characteristics are shown in Table 1. The median patient age is 49 years. The conditioning regimen was myeloablative in 58% and RIC in 42%. PTCy was given either alone (14%), with a calcineurin inhibitor alone (30%), or with the addition of mycophenolate mofetil (34%) in most patients. PTCy was used more recently than in the standard prophylaxis cohort (median year 2017 compared to 2014) and more often with a BM stem cell source (18% compared with 11%).

#### Transplantation outcomes

Transplantation outcomes are presented in Table 1. The incidence of acute GVHD grade II-IV and III-IV at day +180 after SCT was 22.8% (95% CI, 19.2–26.6) and 6.2% (95% CI, 4.3–8.6), respectively. The incidence of all grades and extensive grade chronic GVHD at 2 years after SCT was 35.5% (95% CI, 30.7–40.2) and 17.7% (95% CI, 14.0–21.7), respectively. The 2-year OS was 69.7% (95% CI, 64.9–74.9). The incidence of relapse and NRM at 2 years after allogeneic SCT were 27.1% (95% CI, 22.6–31.9) and 9.3% (95% CI, 6.6–12.5), respectively.

#### Impact of GvHD on survival outcomes

The Cox multivariate model showed that acute GVHD grade II-IV had no statistically significant impact on the incidence of relapse (HR 1.37, *P* = 0.15, Table 4), but was associated with higher NRM, (HR 3.34, *P* < 0.001) and lower OS (HR 1.92, *P* = 0.001). The number of patients in this group did not allow analysis of the different impact of grade II and grade III-IV acute GVHD as in the larger standard GVHD prophylaxis group.

**Table 4:** Prognostic factors for SCT outcomes after PTCY- based prophylaxis.

	Relapse		NRM		OS	
Factor	HR	*P* value	HR	*P* value	HR	*P* value
Acute GVHD						
grade II-IV	1.37 [0.89–2.12]	0.15	3.34 [1.7–6.56]	<0.001	1.92 [1.3–2.85]	0.001
Chronic GVHD	0.99 [0.6–1.63]	0.98	1.11 [0.44–2.78]	0.83	0.73 [0.45–1.17]	0.19
Age	0.98 [0.97–0.99]	0.007	1.07 [1.03–1.11]	<0.001	1.01 [1–1.03]	0.14
Year of SCT	1.04 [0.94–1.14]	0.46	0.84 [0.71–0.99]	0.039	0.98 [0.89–1.07]	0.64
Donor (MUD)	0.68 [0.45–1.01]	0.057	1.9 [0.95–3.81]	0.07	0.94 [0.65–1.38]	0.77
Stem cell source (PBSC)	0.86 [0.51–1.46]	0.58	1.36 [0.45–4.07]	0.58	1.12 [0.64–1.95]	0.7
Status at SCT (CR2)	1.03 [0.54–1.94]	0.94	1.47 [0.6–3.6]	0.4	1.06 [0.59–1.9]	0.85
Cytogenetics (adverse)	2.4 [1.54–3.75]	<0.001	1.17 [0.49–2.78]	0.72	1.93 [1.26–2.95]	0.002
Gender combination (other than F→M)	1.19 [0.71–2]	0.51	0.41 [0.21–0.82]	0.011	0.75 [0.48–1.16]	0.19
KS (<90)	0.9 [0.57–1.42]	0.66	1.26 [0.62–2.57]	0.52	0.93 [0.6–1.43]	0.73
Conditioning (MAC)	0.57 [0.38–0.86]	0.007	2.79 [1.28–6.06]	0.01	1.02 [0.68–1.53]	0.93

Multivariate analysis using acute GVHD and chronic GVHD as time-dependent factors. Abbreviations: As in Tables 1–3.

The Cox multivariate model showed that chronic GVHD of all grades was associated with a similar incidence of relapse (HR 0.99, *P* = 0.98, Table 4), NRM (HR 1.11, *P* = 0.83) and OS (HR 0.73, *P* = 0.19). Similarly, the number of patients did not allow analysis if the impact of the different grades of chronic GVHD. Interestingly, only adverse cytogenetics was a poor prognostic factor for OS, in addition to the poor prognosis associated with acute GVHD in the PTCy cohort (Table 4). A center effect was not detected in any of the analyses.

## Discussion

In this study, we examined the association of GVL and GVHD in two separate cohorts based on the regimen used for GVHD prophylaxis in adult patients with de-novo AML given a first allogeneic SCT from HLA-matched donors in CR1/2. The overall outcome in the modern era of SCT seems markedly improved with a 2-year OS approaching 70%. A marked reduction of NRM to 12% was observed, while relapse rates are still significant. In the first large group of more than 12,000 patients given standard calcineurin-based GVHD prophylaxis, we observed a strong association between these immune effects. Patients who had acute GVHD or extensive chronic GVHD had a reduced risk of relapse (HR 0.85 and 0.76, respectively). However, since this was also associated with a higher risk of NRM, the survival of these patients was reduced. This historical association reported in the early era of SCT [1–3] remains in the more recent era with modern SCT techniques. It was even more significant than previously reported in patients with AML [11, 12]. This observation may be related to the better selection of patients for SCT with deeper remissions where the GVL effect may be more prominent. In addition, better treatment of GVHD may have reduced the opposing effect of GVHD on survival. Several novel treatments have been introduced for the treatment of acute and chronic GVHD including ibrutinib, ruxolitinib, and belumosudil. The EBMT database does not include data on the agents used to treat GVHD, however, these agents were not approved yet during the study period, so we expect that only a minority of patients were given these agents. However, other supportive care techniques have significantly improved as reflected by the low NRM in this study. Interestingly, the strong GVHD/ GVL effect was not affected by the use of in-vivo T-cell depletion or in any subgroup of patient characteristics. Similar observations were observed more recently in large registry cohorts of patients with secondary AML [22] and patients with acute lymphoblastic leukemia [23] also transplanted in the modern era.

PTCy is increasingly being used for GVHD prophylaxis. It was shown to nullify the effect of HLA mismatch between recipients and donors allowing the safe expansion of the donor pool to haploidentical and HLA-mismatched unrelated donor transplants [5–7, 24, 25]. While the dramatic effect of PTCy is still related to the haploidentical setting there is an increased interest in the use of PTCy in HLA-matched transplants as well. The Blood and Marrow Transplantation Clinical Trial Network (BMT-CTN) conducted a phase II trial (BMT CTN 1203) comparing three novel GVHD prophylaxis regimens with the more standard tacrolimus/ methotrexate combination in patients given SCT with RIC [8]. Among these three regimens, the combination of PTCy–tacrolimus– mycophenolate mofetil was the best promising in terms of GVHD-free, relapse-free survival. More recently, the phase III randomized BMT CTN 1703 trial reported that following RIC the PTCy–tacrolimus– mycophenolate mofetil regimen was associated with better GVHD-free, relapse-free survival than tacrolimus/ methotrexate, due to reduction of severe acute GVHD and of chronic GVHD that required treatment with no difference in relapse and survival [9]. This may soon lead to this regimen becoming the standard of care after RIC. The BMT CTN 1301 trial compared single-agent PTCy to other regimens following MAC [26]. Severe chronic GVHD was reduced but this did not translate to better survival. There was a suggestion of lower relapse in the PTCy arm. It seems PTCy is the first regimen to show that more intensive GVHD prophylaxis is not associated with reduced GVL. While this study was not designed to compare GVHD rates after PTCy and non-PTCY groups, they seem to be not markedly different. This may be related to the extensive use of in-vivo T-cell depletion (ATG or alemtuzumab) in the non-PTCy group. In-vivo T-cell depletion is commonly used in Europe, especially in unrelated donor transplants but is used much less often in the US. The BMT-CTN studies did not include any form of in-vivo T-cell depletion agents.

In the second group of patients investigated in the current study given PTCy-based GVHD prophylaxis regimens, we observed a different pattern of GVL / GVHD association than that following the calcineurin-based regimens. We showed that acute GVHD grade II-IV did not reduce relapse risk but there was in fact a borderline statistically significant increased risk of relapse, possibly due to additional immune suppressive therapy given to these patients. NRM was increased and survival was reduced in patients with acute GVHD. Chronic GVHD did not reduce relapse but was relatively well tolerated in the PTCy setting with no increase in NRM or reduced survival. The sample size did not allow for defining the effect of different GVHD grades. We have previously reported similar observations after haploidentical SCT with PTCy [15, 16]. The current  similar report in the HLA-matched setting suggests that it is the use of PTCy rather than the haploidentical donor source that is responsible for the separation of GVHD and GVL. GVL is possibly mediated by GVHD-independent effectors in the PTCy setting. In contrast, the Baltimore group did report a protective effect of mild GVHD post PTCy both in the haplo-identical [27] and in the HLA-matched setting [28]. These discrepancies may be explained by the use of different transplantation platforms, different conditioning regimens, stem cell source (PBSC versus BM), use of ATG, as well as different patient characteristics. Clearly, more studies with biological correlates are required.

Both GVHD and GVL are mediated by donor T-cells and natural killer (NK) cells and therefore occur in parallel. They require the presence of mismatched allo-antigens between the donor and recipient and activation of the immune response. It remains debated if these effects can be separated. Some researchers showed that there are differences between the two effects that can allow targeting of GVL without GVHD [29, 30]. Gale and Fuchs described a potent anti-leukemia effect in haplo-identical SCT with PTCy in patients with no chronic GVHD but could not determine if this is indeed leukemia-specific of a form of undiagnosed GVHD [31]. There are different immune signatures and different T-cell subset reconstitutions related to the mechanism of action of PTCy that may allow the separation between the effects. PTCy impairs the proliferation and cytokine production of alloreactive T-cells but does not completely eradicate them [5, 32]. Thus, it may reduce the progression to a severe form of GVHD and persistence of the GVL effect. PTCy promotes the recovery of regulatory T-cells (T- regs). T-regs, similarly to hematopoietic stem cells are protected from PTCy by a relatively high expression of aldehyde dehydrogenase [33]. They contribute to long-term post-transplant tolerance and prevention of progression to chronic GVHD by limiting T-cell proliferation and downregulation of pro-inflammatory cytokines, while maintaining CD8+ T-cell anti-leukemia activity. These mechanisms are distinct from the mechanism of standard GVHD prophylaxis. The threshold level of T-cells necessary to trigger GVL is lower than that required to trigger GVHD and a lower level of GVHD may be sufficient to reduce the risk of relapse [34]. McCurdy et al. used machine learning techniques to define clinically relevant signatures from multiple immunophenotypic, proteomic, and clinical factors in patients given PTCy after both haploidentical and HLA-matched transplants [35]. Conventional CD4+ T-cell recovery, their activation status, and metabolic signature were associated with acute GVHD and in particular the CXCL9-CXCR3 axis. CD8+ T-cell hypo-responsiveness was less important for protection from acute GVHD. However, NK and CD8+ cells were important in preventing relapse, and a loss of inflammatory gene signature in NK cells and transcriptional exhaustion phenotype in CD8+ T-cells predicted relapse. Interestingly, this was similar for PTCy after matched or haploidentical transplants. Similarly, Zhao et al. also showed a distinct T-cell immune signature after PTCy [36]. In particular, there was remarkable enhancement of multiple inhibitory receptors both on CD4+ and CD8+ cells with reduced response to stimulation, while loss of granzyme and perforin expression on CD8+ cells was associated with relapse.

The current analysis has several limitations. The study was not designed to compare standard GVHD prophylaxis with PTCy but rather to analyze the GVHD/ GVL association separately. Although patient characteristics seem similar, the two groups may not have had equivalent patient and transplant characteristics. In particular, the database does not include the reason for selecting the PTCy regimen over the more standard GVHD prophylaxis. PTCy may have been used more often by certain centers but a center effect was not shown.

Overall outcome seems similar between the groups. The absence of GVHD/GRFS differences between the two groups may call into question that the lack of relapse-protective effects associated with GVHD after PTCy is representative of the PTCy field and randomized studies would be more appropriate for this comparison [9]. The PTCy group was relatively small, not allowing definition of the role of different grades of acute and chronic GVHD in this group. The database did not allow characterization of the different clinical pictures, organ involvement, and response to therapy in the two groups.

In conclusion, GVHD of any type or grade may not be associated with an improved relapse rate after HLA-matched SCT with PTCy and probably offers no survival advantage. Severe forms are associated with higher NRM and lower survival. Future novel strategies for further reducing significant GVHD while preserving GVL are warranted.

## Data Availability

The data are available from the corresponding author upon reasonable request.
